# Endocannabinoid System in Spinocerebellar Ataxia Type-3 and Other Autosomal-Dominant Cerebellar Ataxias: Potential Role in Pathogenesis and Expected Relevance as Neuroprotective Targets

**DOI:** 10.3389/fnmol.2019.00094

**Published:** 2019-04-24

**Authors:** María Gómez-Ruiz, Carmen Rodríguez-Cueto, Eva Luna-Piñel, Mariluz Hernández-Gálvez, Javier Fernández-Ruiz

**Affiliations:** ^1^Departamento de Bioquímica y Biología Molecular, Instituto Universitario de Investigación en Neuroquímica, Facultad de Medicina, Universidad Complutense de Madrid, Madrid, Spain; ^2^Departamento de Psicobiología, Facultad de Psicología, Universidad Complutense de Madrid, Madrid, Spain; ^3^Centro de Investigación Biomédica en Red de Enfermedades Neurodegenerativas, Madrid, Spain; ^4^Instituto Ramón y Cajal de Investigación Sanitaria, Madrid, Spain

**Keywords:** cannabinoids, endocannabinoid system, autosomal-dominant inherited ataxias, motor incoordination, neuroprotection

## Abstract

Spinocerebellar ataxias (SCAs) are a group of hereditary and progressive neurological disorders characterized by a loss of balance and motor coordination typically associated with cerebellar atrophy. The most prevalent SCA types are all polyQ disorders like Huntington’s disease, sharing the most relevant events in pathogenesis with this basal ganglia disorder, but with most of the damage concentrated in cerebellar neurons, and in their afferent and efferent connections (e.g., brainstem nuclei). SCAs have no cure and effective symptom-alleviating and disease-modifying therapies are not currently available. However, based on results obtained in studies conducted in murine models and information derived from analyses in *post-mortem* tissue samples from patients, which show notably higher levels of CB_1_ receptors found in different cerebellar neuronal subpopulations, the blockade of these receptors has been proposed for acutely modulating motor incoordination in cerebellar ataxias, whereas their chronic activation has been proposed for preserving specific neuronal losses. Additional studies in *post-mortem* tissues from SCA patients have also demonstrated elevated levels of CB_2_ receptors in Purkinje neurons as well as in glial elements in the granular layer and in the cerebellar white matter, with a similar profile found for endocannabinoid hydrolyzing enzymes, then suggesting that activating CB_2_ receptors and/or inhibiting these enzymes may also serve to develop cannabinoid-based neuroprotective therapies. The present review will address both aspects. On one hand, the endocannabinoid system becomes dysregulated in the cerebellum and also in other CNS structures (e.g., brainstem, basal ganglia) in SCAs, which may contribute to the progression of pathogenic events in these diseases. On the other hand, these endocannabinoid alterations may be pharmacologically corrected or enhanced, and this may have therapeutic consequences, either alleviating specific symptoms or eliciting neuroprotective effects, an objective presently under investigation.

## Brief Overview on Cannabinoids as Neuroprotectants

**FIGURE 1:**
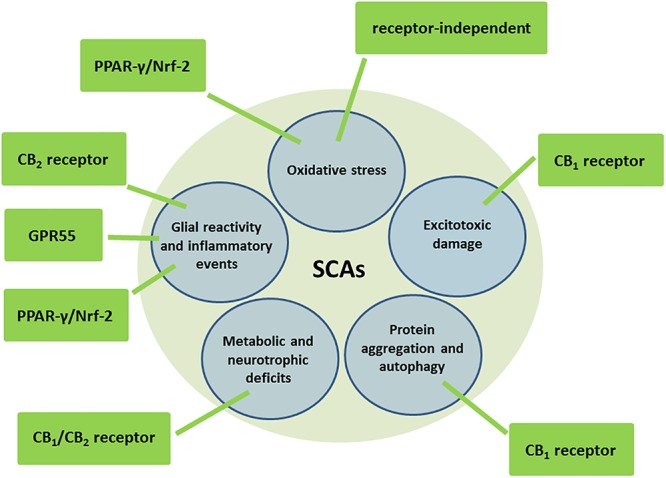
Molecular and cellular mechanisms potentially involved in the neuroprotective effects of cannabinoids in autosomal-dominant SCAs.

Cannabinoids are a family of pleiotropic compounds identified for the first time in the cannabis plant (phytocannabinoids) but which can also be obtained in the laboratory (synthetic cannabinoids) and are present in animal tissues (endocannabinoids). In general, they are active at the so-called endocannabinoid system, a modulatory system involved in the maintenance of cell and tissue homeostasis (reviewed recently in García-Arencibia et al., 2018). Given the importance of this homeostatic role in the cell integrity, the endocannabinoid system has been strongly associated with the survival/death cell decision, so that those cannabinoids capable of targeting and activating/inhibiting specific endocannabinoid elements are able to exert both cytoprotective/cytorepair properties (reviewed recently in Fernández-Ruiz, 2018) and proapoptotic/antitumoral effects (reviewed in Velasco et al., 2016). The cytoprotective/cytorepair properties of cannabinoids have been broadly investigated in the Central Nervous System (CNS) in diseases associated with nerve cell injury, demonstrating that cannabinoids display a broad-spectrum neuroprotective profile (antiexcitotoxic, antioxidant, anti-inflammatory, pro-autophagy, and pro-neurogenic effects; reviewed recently in Aymerich et al., 2018), an important advantage in neurodegenerative disorders in which the damage to neurons and glial cells is provoked by a concerted action of different cytotoxic insults (e.g., excitotoxicity, oxidative damage, glial reactivity/inflammatory events, protein aggregation, reduced neurogenesis). This is possibly because, compared to other neuroprotectants, the effects of cannabinoids may be exerted through quite diverse and complementary cellular and molecular mechanisms, for example by activating cannabinoid type-1 (CB_1_) or type-2 (CB_2_) receptors, but also peroxisome proliferator-activated receptors (PPARs) or GPR55, or even through receptors/targets completely outside the endocannabinoid system (Fernández-Ruiz, 2018). In this way, a single cannabinoid or a combination of cannabinoids acting in conjunction might, at the same time, normalize glutamate homeostasis (CB_1_ receptor-mediated), reduce oxidative stress (receptor-independent and/or PPAR-γ/Nrf-2-mediated mechanisms) and glial activation (CB_2_, PPAR-γ/Nrf-2 and/or GPR55 receptor-mediated), promote autophagy to eliminate protein aggregates (CB_1_ receptor-mediated), and enhance metabolic and neurotrophic support (CB_1_/CB_2_ receptor-mediated) (see Figure 1; reviewed in Fernández-Ruiz, 2018). They can also promote the proliferation, maturation and differentiation of neural progenitor cells, which may also facilitate nerve cell replacement, a critical issue in neurodegenerative disorders that are frequently diagnosed when neuronal damage is already important (reviewed recently in Fernández-Ruiz, 2018). Such a broad-spectrum neuroprotective/neurorepair profile of cannabinoids is facilitated by the fact that those endocannabinoid and non-endocannabinoid targets activated/inhibited by these compounds are located in CNS substrates and structures that play a crucial role in the preservation, rescue, repair and replacement of neurons, in concordance with the above-mentioned neuroprotective function assigned to the endocannabinoid system (Fernández-Ruiz, 2018; García-Arencibia et al., 2018).

These neuroprotective and to a lesser extent, neurorepairing properties of cannabinoids are being extensively investigated in acute (e.g., ischemia, brain trauma, spinal lesion) and chronic neurodegenerative disorders such as Alzheimer’s disease, Parkinson’s disease, Huntington’s chorea, and more recently, amyotrophic lateral sclerosis (reviewed in Fernández-Ruiz et al., 2015; Arevalo-Martin et al., 2016; Aymerich et al., 2018), with variable results but, in some cases, driving the issue even to the clinical scenario although still without positive results (López-Sendón et al., 2016). Additional neurodegenerative disorders, such as spinocerebellar ataxias (SCAs), have recently attracted certain interest for the possibility to receive benefits for novel neuroprotective treatments in a group of disorders with a still poor therapeutic outcome (Stephens, 2016). To review this interest and its supporting experimental evidence is the main objective of this article.

## Spinocerebellar Ataxias

Cerebellar ataxias are a group of progressive neurological disorders characterized by a loss of balance and motor coordination typically associated with cerebellar dysfunction and/or atrophy (Rossi et al., 2014). They can be acquired (derived from brain traumas, infections, ischemia, alcohol misuse or other causes; Klockgether, 2011) or presented as hereditary forms, which include (i) autosomal-dominant SCAs, in which the most prevalent cases belong to the family of polyglutaminopathies (polyQ) (Durr, 2010; Klockgether, 2011; Seidel et al., 2012; Rüb et al., 2013; Pulst, 2016; Buijsen et al., 2019; see Table 1 for a summary of major characteristics of SCAs being polyQ disorders), that also includes Huntington’s disease (Ghosh and Tabrizi, 2018); and (ii) autosomal-recessive ataxias (e.g., Friedreich’s ataxia) (Klockgether, 2011).

**Table 1:** Some examples of polyQ disorders including most important SCAs and Huntington’s disease, with details of the protein affected, the differences in the length of the normal and pathological expansion, and the major neuropathological and clinical signs.

PolyQ disorders	Affected protein	polyQ expansion		Major neuropathological and clinical signs
		Normal	Pathological	
				Degeneration affecting:
SCA-1	Ataxin-1	6–39	≥41	• Cerebellum and brainstem (ataxia)
SCA-2	Ataxin-2	14–32	≥34	• Optic nerve (ophthalmoplegia and visual loss)
SCA-3	Ataxin-3	12–40	≥62	• Basal ganglia (parkinsonian signs)
				• Cerebral cortex (cognitive impairment)
SCA-17	TATA-binding protein	25–43	≥45	• Peripheral nerves (neuropathy)
				• Nuclear inclusions preferentially

SCA-6	CACNA 1A	4–18	≥21	• Cerebellar degeneration only (ataxia)
				• Cytoplasmic inclusions

SCA-7	Ataxin-7	7-18	≥38	• Cerebellar degeneration (ataxia)
				• Retinopathy (visual loss)

Huntington’s disease	Huntingtin	6–35	≥40	• Striatal degeneration (chorea)
				• Cortical degeneration (dementia)

As mentioned above, in this review we will concentrate on SCAs, and in particular, on SCA-3 or Machado-Joseph disease, which is the most prevalent form of SCAs and one of the diseases that has been reproduced in laboratory animals (e.g., mice, fishes, flies, nematodes) with relative success. SCA-3 is a polyQ disorder caused by excessive CAG repeats leading to the expansion of a polyglutamine tract in ataxin-3, a protein identified in the study of the disease, whose normal function is related to many cellular functions, in particular to the control of ubiquitination/deubiquitination protein balance (Rüb et al., 2013). In SCA-3, ataxin-3 may form protein aggregates (predominantly intranuclear inclusions)—derived from misfolding, failed or incomplete proteolysis, deposition and aggregation (Paulson, 2012)—which damage several cell organelles; for example the nucleus, thus eliciting transcriptional dysregulation, but also the mitochondria and others, thus producing dysregulation in calcium homeostasis, oxidative stress, excitotoxic events and glial reactivity/local inflammation (Matilla-Dueñas et al., 2012; Orr, 2012). All these events would contribute together to kill those neuronal subpopulations that are more vulnerable to mutant ataxin-3 (despite the ubiquitous expression of this protein), which are located mainly in the cerebellum (dentate nucleus), but also in the brainstem (pontine nuclei) and in the basal ganglia (striatum) (Taroni and DiDonato, 2004; Koeppen, 2005, 2018; Fratkin and Vig, 2012; Matilla-Dueñas, 2012; Koeppen et al., 2013; Seidel et al., 2016). This selectivity in neuronal death explains that clinical symptoms in SCA-3 patients are diverse and heterogeneous. They can include progressive loss of motor coordination and gait anomalies derived from primary cerebellar and brainstem degeneration (Riess et al., 2008; Matilla-Dueñas, 2012; Koeppen, 2018), but also other symptoms related to muscle atrophy (e.g., peripheral amyotrophy), retinal damage (e.g., ophtalmoparesis), pyramidal signs (e.g., spasticity, hyperreflexia), and basal ganglia deterioration (e.g., rigidity, dystonia) (Matilla-Dueñas et al., 2012; Jacobi et al., 2013; Rüb et al., 2013; Koeppen, 2018; see Table 1).

Spinocerebellar ataxia type-3, better in addition to the other SCAs as well as the remaining types of ataxias, are unfortunately diseases with no cure and lacking effective treatments both for alleviating clinical symptoms, and in particular, for modifying disease progression (Rüb et al., 2013; Paulson et al., 2017). However, novel strategies are currently being investigated; for example (i) silencing strategies based on ShRNAs, siRNAs, specific miRNAs or antisense oligonucleotides to reduce the translation of the mutated protein (Alves et al., 2010; Conceição et al., 2016; Moore et al., 2017; Matos et al., 2019); (ii) molecular (e.g., Hsp104) or chemical chaperones, or inhibition of the proteolytic cleavage of ataxin-3 (e.g., calpain inhibitors) to reduce protein aggregation (Matos et al., 2019); (iii) autophagy activators to eliminate protein aggregates (Menzies et al., 2010; Nascimento-Ferreira et al., 2013; Silva-Fernandes et al., 2014); and (iv) inhibitors of histone deacetylases to attenuate the transcriptional dysregulation caused by intranuclear inclusion bodies (Chou et al., 2011; Lei et al., 2016), as well as additional strategies to normalize other cellular processes (e.g., ubiquitin-proteasome system, mitochondrial function) affected by these protein aggregates (Matos et al., 2019).

Studies carried out in experimental models of SCA-3, as well as analyses of *post-mortem* tissue samples obtained from SCA patients, proved the occurrence of elevated levels of CB_1_ receptors in different cerebellar neuronal subpopulations (Rodríguez-Cueto et al., 2014a, 2016; see below and Table 2). Given that the activation of cerebellar CB_1_ receptors has been associated with signs of motor incoordination (DeSanty and Dar, 2001; Patel and Hillard, 2001), the blockade of these receptors (and similar pharmacological interventions) has been proposed for attenuating this cardinal sign of cerebellar ataxias (reviewed in Stephens, 2016). Additional analyses carried out in *post-mortem* tissues from SCA patients have also demonstrated elevated levels of CB_2_ receptors in cerebellar neurons (e.g., Purkinje cells, neurons of the dentate nucleus) and in glial elements of the cerebellar granular layer and the white matter (Rodríguez-Cueto et al., 2014a; see below and Table 2), accompanied by similar elevations in endocannabinoid hydrolyzing enzymes (Rodríguez-Cueto et al., 2014b; see below and Table 2). Such alterations support that activating CB_2_ receptors and/or inhibiting these enzymes may serve to develop potential cannabinoid-based disease-modifying therapies, which may also include compounds activating the CB_1_ receptors to limit, for example, excitotoxic damage. In the next two sections, we will review the evidence collected so far that demonstrates how the endocannabinoid system is altered in SCAs, in particular SCA-3, in the different CNS structures affected in these diseases, and how these alterations may contribute to SCA pathogenesis by aggravating excitotoxicity, oxidative stress, protein deposition and aggregation, and glial reactivity. We will lastly discuss the proposals that have been formulated to generate cannabinoid-based therapies for SCAs based on the correction of potential endocannabinoid dysregulations and/or on the enhancement of possible endocannabinoid-based endogenous protective responses.

**Table 2:** Summary of changes in endocannabinoid elements in autosomal-dominant SCAs observed in experiments with tissue samples from both patients and SCA-3 transgenic mice.

Endocannabinoid elements	CNS structures and sources of tissues	Major observations and findings (Rodríguez-Cueto et al., 2014a,b, 2016, 2017)
CB_1_ receptors	Cerebellum (patients)	↑↑↑ in surviving neurons (Purkinje cells, dentate nucleus)
		↑↑↑ in glial cells (granular layer and white matter areas)
	Cerebellum (SCA-3 mice)	↑↑↑ in the Purkinje cell layer (terminals of basket cells)
		↓↓↓ in neurons of the dentate nucleus
	Brainstem (SCA-3 mice)	No changes detected
	Basal ganglia (SCA-3 mice)	↓↓↓ in striatal afferent and efferent neurons

CB_2_ receptors	Cerebellum (patients)	↑↑↑ in glial cells (granular layer and white matter areas)
		↑↑↑ in surviving neurons (Purkinje cells, dentate nucleus)
	Cerebellum (SCA-3 mice)	No changes detected
	Brainstem (SCA-3 mice)	No changes detected
	Basal ganglia (SCA-3 mice)	No changes detected

FAAH enzyme	Cerebellum (patients)	↑↑↑ in surviving neurons (Purkinje cells)
		↑↑↑ in glial cells (granular layer and white matter areas)
	Cerebellum (SCA-3 mice)	↑↑↑ in Purkinje cells and in white matter areas.
		No changes detected in the dentate nucleus
	Brainstem (SCA-3 mice)	↑↑↑ in the pontine nuclei
	Basal ganglia (SCA-3 mice)	↑↑↑ in the striatum

MAGL enzyme	Cerebellum (patients)	↑↑↑ in surviving neurons (Purkinje cells, dentate nucleus)
		↑↑↑ in glial cells (granular layer and white matter areas)
	Cerebellum (SCA-3 mice)	No changes detected
	Brainstem (SCA-3 mice)	No changes detected
	Basal ganglia (SCA-3 mice)	No changes detected

Levels of endocannabinoids and related lipids	Cerebellum (patients)	Not investigated
	Cerebellum (SCA-3 mice)	No changes detected
	Brainstem (SCA-3 mice)	↓↓↓ in anandamide and oleylethanolamide
	Basal ganglia (SCA-3 mice)	No changes detected

## Status of the Endocannabinoid System in SCAs

Only a few recent studies have investigated whether specific elements of the endocannabinoid system are altered in CNS structures that have been associated with the clinical signs of SCAs, namely the cerebellum, brainstem, and basal ganglia (Rodríguez-Cueto et al., 2014a,b, 2016, 2017). As mentioned above, to investigate this possibility is important both for elucidating whether these alterations may contribute to SCA pathogenesis and for developing potential cannabinoid-based therapies that may correct these signs, and eventually, to delay/arrest disease progression in SCAs.

### Studies in the *Post-mortem* Cerebellum of SCA Patients

The first studies to determine the status of the endocannabinoid system consisted of analyses of some key endocannabinoid elements (e.g., CB_1_ receptor, CB_2_ receptor, fatty acid amide hydrolase (FAAH), monoacylglycerol lipase (MAGL)) in the *post-mortem* cerebellum of different SCA patients compared to control subjects (Rodríguez-Cueto et al., 2014a,b; see Table 2 for a summary of these endocannabinoid elements in SCA patients). These studies demonstrated first elevations in the two major cannabinoid receptors, CB_1_ and CB_2_, in different cerebellar areas, including some neuronal subpopulations which, in the case of CB_2_ receptors, are not usually located or their expression is too weak, such as basket cells, Purkinje cells, and in particular, neurons of the dentate nucleus (Rodríguez-Cueto et al., 2014a). It is important to remark that these elevations of CB_1_/CB_2_ receptors in cerebellar neurons were detected in those cells that survived the degeneration before patient death, therefore the elevations were evident comparing individual cells of SCA patients and control subjects. However, when these data were referred to the whole structure, the total levels of these receptors may result in being reduced due to the marked losses of Purkinje cells and neurons of the dentate nucleus occurring in SCAs.

The identification of CB_1_ receptors in neuronal substrates is normal; however, this is not the case for the CB_2_ receptor, whose cellular distribution in the CNS is predominantly glial (Fernández-Ruiz et al., 2007, 2015; Aymerich et al., 2018), although relatively recent studies have identified CB_2_ receptors also in some restricted neuronal subpopulations in different CNS structures (e.g., cerebellar granular cells, brainstem neurons, pallidothalamic and nigrostriatal neurons, cortical pyramidal neurons and others, although this may depend on the animal species analyzed; reviewed in Aymerich et al., 2018). In our study in SCA patients, CB_2_ receptors were identified (and found to be elevated when measuring individual cells) in similar neuronal substrates to CB_1_ receptors, including basket neurons, Purkinje cells and neurons of the dentate nucleus (Rodríguez-Cueto et al., 2014a). However, major cellular substrates of CB_2_ receptors in the cerebellum of SCA patients were also in our study, including reactive microglia, macrophages and astrocytes located in the granular layer and in the white matter area of the folia and also surrounding the dentate nucleus, showing always elevated levels in patients compared to controls (Rodríguez-Cueto et al., 2014a).

In a follow-up study, using immunostaining and double-labeling immunofluorescence, endocannabinoid-inactivating enzymes, e.g., FAAH, MAGL, were detected in the granular layer, Purkinje cells, neurons of the dentate nucleus and areas of white matter at levels notably higher in SCA patients (Rodríguez-Cueto et al., 2014b), then supporting a reduction of endocannabinoid levels in these areas due to an excessive degradation by these enzymes. Such changes may be interpreted as apparently contradictory with the observation of elevated levels of CB_1_/CB_2_ receptors. It is possible, however, that the elevation of cannabinoid receptors may be the primary effect, which would secondarily generate a compensatory response elevating hydrolyzing enzymes with the objective to reduce the availability of endocannabinoids capable of activating CB_1_/CB_2_ receptors, then lowering the elevated signaling depending on these receptors in the cerebellum of SCAs (Rodríguez-Cueto et al., 2014a,b). However, the opposite interpretation may also be valid, with a primary effect in the elevation of FAAH/MAGL, resulting in low levels of endocannabinoids, which would activate a classic response in ligand-receptor regulation consisting of upregulating CB_1_/CB_2_ receptors (or increasing their activity/signaling) with the purpose of opposing the elevated FAAH/MAGL-dependent degradation of endocannabinoids (Rodríguez-Cueto et al., 2014a,b). Therefore, to determine whether these changes in enzymes are a primary event or a secondary effect aimed at compensating for elevated CB_1_/CB_2_ receptor signaling derived from their elevated receptor levels, is an important objective that we are presently investigating in experimental models of SCA-3 (see below).

### Studies in an Animal Model of SCA-3

The changes detected in the endocannabinoid signaling in *post-mortem* tissues from SCA patients corresponded to a very advanced stage of the disease, when patients died, and they do not tell us whether they also exist (or are different) during the presymptomatic, early symptomatic and stable symptomatic stages of disease progression. Such clarification is critical to determine whether the changes in endocannabinoid signals may be part of the pathogenic events contributing to the progression of symptoms and of the degenerative process. Such types of studies need to be carried out in experimental models of autosomal-dominant inherited ataxias, which recapitulate the major neuropathological characteristics of these disorders, although such models only exist for a few types of SCAs, namely SCA-1, SCA-2, SCA-3, SCA-6, SCA-7, SCA-17, SCA-23 and others (see Gould, 2012; Cendelin, 2014; Hoxha et al., 2018, for review). We had the opportunity to work with a transgenic mouse model of SCA-3 generated by Silva-Fernandes et al. (2014), which reproduces many of the neurological and neuropathological signs of the disease, following a gradual and progressive pattern with expression of mutant ataxin-3 at near endogenous levels. We used these mice for demonstrating a possible relation of specific symptoms and neuropathological lesions observed in SCA-3 with dysregulation of the endocannabinoid system in two of the most-affected CNS structures in SCA-3: the cerebellum and brainstem (see Table 2 for a summary of those changes in endocannabinoid elements seen in these two structures of SCA-3 transgenic mice). If this were the case, such dysregulation may be pharmacologically corrected, which would have benefits for symptoms, and in particular, for disease progression. This may also include enhancing those changes that represent an adaptive response involving the endocannabinoid system, with the objective of restoring cell and tissue homeostasis as described in other neurodegenerative disorders (Di Marzo et al., 2015). Our studies confirmed both possibilities (Rodríguez-Cueto et al., 2016). On one hand, we found elevated CB_1_ receptor levels in the Purkinje cell layer of SCA-3 transgenic mice, with the receptors located predominantly in terminals of basket cells, and the opposite response in the dentate nucleus, although this reduction was possibly due to the marked neuronal losses seen in this nucleus in SCA-3 transgenic mice (Rodríguez-Cueto et al., 2016). No changes were detected in CB_1_ receptors in the brainstem nuclei of SCA-3 transgenic mice (Rodríguez-Cueto et al., 2016). On the other hand, we also detected elevated FAAH levels and immunostaining, as well as FAAH-mediated degradation of endocannabinoids, in some cerebellar structures (e.g., Purkinje cells, areas of white matter) and in particular, in the pontine nuclei of the brainstem (Rodríguez-Cueto et al., 2016). It is interesting to remark that these changes (e.g., elevated CB_1_ receptor and FAAH levels) were also found in cerebellar nuclei in the studies using *post-mortem* tissues of SCA patients (Rodríguez-Cueto et al., 2014a,b), as described previously.

As mentioned above, the neuropathology of SCAs, in particular in those with higher prevalence such as SCA-3, is not restricted to the cerebellum and brainstem, being also evident in other CNS structures, in particular the basal ganglia (mainly the striatum) and also certain cortical areas, although these latter areas have not yet been investigated in relation with cannabinoids. As mentioned above, the affectation of these cortical and subcortical structures explains why patients develop non-cerebellar extrapyramidal signs (e.g., rigidity, dystonia) and even dementia (reviewed in Matilla-Dueñas, 2012; Jacobi et al., 2013; Rüb et al., 2013; Koeppen, 2018), in addition to primary cerebellar symptoms such as progressive loss of motor coordination, abnormal gait and others. Such non-cerebellar signs (e.g., limb clasping as a sign of dystonia) may also be reproduced in experimental models of SCA-3 and associated with the changes in the endocannabinoid system in the striatum. Thus, in a second study carried out in SCA-3 transgenic mice (Rodríguez-Cueto et al., 2017; see Table 2 for a summary of those changes in endocannabinoid elements seen in the striatum of SCA-3 transgenic mice), we detected a reduction of CB_1_ receptor levels associated with an elevation of FAAH levels in the striatum of SCA-3 transgenic mice. The reduction of CB_1_ receptor levels was found in both striatal afferent and efferent neurons—in this last case, being associated with the losses of striatal projection neurons found in the SCA-3 transgenic mice (Rodríguez-Cueto et al., 2017). As will be discussed below, reductions in CB_1_ receptor signaling may obviously aggravate excitotoxicity, which may explain the losses of striatal projection neurons in these mice, a fact also aggravated by the elevation detected in the FAAH enzyme also in the striatum, which would eventually reduce the levels of endocannabinoid ligands acting at the CB_1_ receptor (Rodríguez-Cueto et al., 2017).

Our studies in SCA-3 transgenic mice also included the analysis of the CB_2_ receptor in the three most-affected CNS structures (Rodríguez-Cueto et al., 2016, 2017), following the data collected in SCA patients which demonstrated elevated levels of this receptor in the cerebellum (Rodríguez-Cueto et al., 2014a). As mentioned above, this receptor is currently located in the CNS in glial elements, in particular when they become reactive (Fernández-Ruiz et al., 2007, 2015), but in our studies in SCA patients, it was found to be elevated not only in glial elements, but also in neuronal subpopulations (Rodríguez-Cueto et al., 2014a). However, our studies in SCA-3 transgenic mice proved that CB_2_ receptors do not appear to exert an important role in relation to the progression of the pathological phenotype in these mice, nor do they appear to be relevant as a potential neuroprotective and anti-inflammatory target (Rodríguez-Cueto et al., 2016, 2017). Such an observation is not strange as neurodegeneration in some SCAs (including particularly SCA-3) does not appear to be associated with marked glial activation and inflammatory events (Rüb et al., 2013). Lastly, we also measured the levels of endocannabinoids and related signaling lipids in the most affected CNS structures in SCA-3 transgenic mice, but our data indicated that they remain normal (Rodríguez-Cueto et al., 2016, 2017; see Table 2), except for an early and modest reduction in anandamide and oleylethanolamide detected in the brainstem (Rodríguez-Cueto et al., 2016; see Table 2).

### Possible Implications Derived From These Endocannabinoid Alterations

Some important consequences may derive from the data described in the above subsections supporting a possible dysregulation of the endocannabinoid system in CNS structures most affected in SCAs, in particular SCA-3. On one hand, what is clear from these mouse and human data is that the occurrence of such dysregulated endocannabinoid signals is clearly dependent on cellular context, as they are not similar in the different structures investigated. On the other hand, these changes may have an instrumental value, namely they may contribute to enhance specific events that during SCA pathogenesis lead to neuronal degeneration, so that it is possible that the pharmacological manipulation of these changes (inhibition or enhancement) may serve to slow disease progression, as has been indicated above. For example, the losses of CB_1_ receptors may aggravate excitotoxicity, given the role that this receptor plays in the control of glutamate homeostasis, and such aggravation may accelerate neuronal injury, with the opposite, e.g., elevated CB_1_ receptor signaling associated with no neuronal death, being also evident (Fernández-Ruiz et al., 2015). Such situations were found in our study in SCA-3 transgenic mice with the changes observed in CB_1_ receptors in two key cerebellar areas: the Purkinje cell layer and the dentate nucleus (Rodríguez-Cueto et al., 2016). We found no losses of Purkinje cells or of other neuronal subpopulations located in cerebellar layers, associated with CB_1_ receptors that were preserved, and even elevated, in the Purkinje cell layer (Rodríguez-Cueto et al., 2016). The opposite situation appears to occur in the deep cerebellar nuclei, with a reduction in CB_1_ receptors in neurons of the dentate nucleus and also in terminals contacting such neurons, which experienced an important level of cell death in SCA-3 transgenic mice (Rodríguez-Cueto et al., 2016). Similar conclusions were also derived from our data of FAAH levels, in particular in the pontine nuclei of the brainstem, in which we detected an elevation of this enzyme paralleled by reduced levels of endocannabinoids (Rodríguez-Cueto et al., 2016), which may facilitate the neuronal losses found in the pontine nuclei due to the reduction in the protective role on cellular homeostasis exerted by these signaling lipids. As mentioned above, the same situation—even worsened—was evident in the striatum of SCA-3 transgenic mice, in which we detected both elevated FAAH levels (leading to reduced endocannabinoid availability) and reduced CB_1_ receptor signaling, with both events being likely responsible for the losses of striatal projection neurons found in these SCA-3 transgenic mice (Rodríguez-Cueto et al., 2017).

Another important consequence derived from our patient and mouse data allows relating the changes in the endocannabinoid system to the appearance of specific neurological symptoms in this disease (e.g., ataxia). This is the case of the elevated CB_1_ receptor levels found in the Purkinje cell layer (predominantly in terminals of the basket cells) in tissues obtained from both SCA patients (Rodríguez-Cueto et al., 2014a) and SCA-3 transgenic mice (Rodríguez-Cueto et al., 2016), which may have a prompt influence in the occurrence of ataxia and motor incoordination, independently of the potential role of this receptor in slowing the progression of the disease. In fact, signs of limb ataxia have been described in naïve animals simply after the activation of CB_1_ receptors in different pharmacological studies (DeSanty and Dar, 2001; Patel and Hillard, 2001). The same relation between CB_1_ receptor activation and signs of motor incoordination has been described in studies carried out with the first neurological disorder associated with mutations in a member of the endocannabinoid gene family, the gene encoding the catabolic enzyme α/β-hydrolase domain-containing 12 (ABHD12) enzyme (Fiskerstrand et al., 2010; Chen et al., 2013), which has the highest expression in microglial cells (Savinainen et al., 2012). The mutations found in this enzyme lead to a disease called PHARC, whose acronym recapitulates the different neuropathological conditions found in the disease (PHARC = polyneuropathy, hearing loss, ataxia, retinitis pigmentosa and cataract). The mutations originate a loss-of-function in this enzyme, which would elevate 2-AG levels in the CNS (at least in those areas having lower MAGL activity), which, through an excess of CB_1_ receptor activation, could explain the appearance of ataxia. In support of this idea, the genetic ablation of this enzyme in mice resulted in the appearance of ataxia and muscle weakness, associated with microglial activation in several CNS structures (Blankman et al., 2013).

By contrast with our studies carried out in SCA-3 transgenic mice that relate elevated CB_1_ receptor signaling in the cerebellum to signs of ataxia, other studies showed apparently opposite results. For example, Gary Stephens and coworkers, using the mouse “ducky” model of cerebellar ataxia (*du*^2J^/*du*^2J^; mice with deficits in auxiliary α2δ2 subunits of the voltage-dependent calcium channel), described a deficient presynaptic CB_1_ receptor signaling in the cerebellar cortex (Wang et al., 2013). They interpreted these CB_1_ receptor defects as a necessary contribution to the development of a progressive ataxic phenotype, an observation that is also of interest for the design of cannabinoid-based therapies to attenuate ataxias (Stephens, 2016). However, it is important to remark that this murine model may recapitulate better SCA-6, which is the SCA type with greater deterioration of Purkinje cells (as also found in the *du*^2J^/*du*^2J^ mouse “ducky” model of cerebellar ataxia; Wang et al., 2013) derived from the naturally occurring mutations in the α1A subunit of the same channel.

## Conclusion and Perspectives for a Future Development of Pharmacological Treatments with Cannabinoids Useful for SCAs

In summary, the studies conducted so far to determine the status of the endocannabinoid signaling in those CNS structures that were most affected in SCAs, and in particular in SCA-3, confirm the possible existence of a dysregulation in the endocannabinoid system, affecting predominantly the CB_1_ receptors and the FAAH enzyme in these CNS structures. Such dysregulation has also been found in Huntington’s disease, which is the most prevalent polyQ disorder, also affecting the CB_1_ receptor and the FAAH enzyme in the striatum (reviewed in Fernández-Ruiz et al., 2015) with changes that are relatively similar to those found in the same structure in the SCA-3 transgenic mice (Rodríguez-Cueto et al., 2017). Considering the experience accumulated in Huntington’s disease (reviewed in Fernández-Ruiz et al., 2015), it appears feasible that a pharmacological manipulation addressed to correct the changes in the endocannabinoid system could be a promising option in SCAs, in particular in SCA-3, which is the type more investigated to date in relation to cannabinoids. Such pharmacological interventions are currently under investigation and include therapies aimed at reducing specific symptoms and/or at slowing down disease progression.

The possibility to develop therapies for alleviating specific symptoms, for example to reduce ataxia, is concentrated, as mentioned above, in the blockade of the elevated CB_1_ receptor signaling detected in the Purkinje cell layer, predominantly in terminals of the basket cells (Rodríguez-Cueto et al., 2014a, 2016). As already indicated, previous pharmacological studies conducted with naïve laboratory animals, not with SCA animals, support the possible benefits of blocking CB_1_ receptor signaling against signs of ataxia and motor incoordination, but these studies were based on investigating the opposite pharmacological intervention, e.g., the activation of CB_1_ receptors generates ataxic signs (DeSanty and Dar, 2001; Patel and Hillard, 2001). However, as also mentioned above, some studies suggested the opposite intervention to treat ataxic signs, e.g., to enhance CB_1_ receptor signaling, based on data showing defects in this signaling in the *du*^2J^/*du*^2J^ mouse “ducky” model of cerebellar ataxia (Wang et al., 2013; Stephens, 2016).

Beyond the treatment of specific neurological symptoms, cannabinoids may also serve in SCAs as disease modifiers given their well-known neuroprotective properties indicated in the first section of this review article (Fernández-Ruiz et al., 2015). This is possibly a better option to reach effective and sustained symptom relief (e.g., reduction in ataxic signs), as described in other neurodegenerative disorders (Fernández-Ruiz et al., 2015), and could be reached with cannabinoids capable of activating the CB_1_ receptor, which may be effective in reducing excitotoxicity (Fernández-Ruiz et al., 2015), and also with those activating the CB_2_ receptor (see below), which may be active against glial reactivity and toxicity (Fernández-Ruiz et al., 2007, 2015). The data obtained in SCA-3 transgenic mice (Rodríguez-Cueto et al., 2016, 2017) may confirm the potential of cannabinoids targeting the CB_1_ receptor, for example for preserving the neurons of the dentate nucleus, whose CB_1_ receptor signaling is hampered (Rodríguez-Cueto et al., 2014a, 2016). The activation of the CB_1_ receptor would be aimed at correcting this dysregulation in the endocannabinoid system, assuming that the dysregulation represents a maladaptive response that contributes to disease progression. The elevation of FAAH-mediated hydrolysis of endocannabinoids found in pontine nuclei of the brainstem (Rodríguez-Cueto et al., 2016) would also be a maladaptive response contributing to disease progression due to the expected reduction in endocannabinoids. Therefore, inhibitors of the enzyme FAAH (and possibly also of other hydrolyzing enzymes), which have been developed in recent years (reviewed by Hwang et al., 2010), may also become prevalent in potential disease-modifying therapies in these disorders due to their capability to enhance the levels of endocannabinoids and their protective function.

The neuroprotective effects of certain cannabinoids may also include enhancing specific responses of this signaling that represent adaptive mechanisms aimed at restoring neuronal homeostasis against different damaging stimuli (Di Marzo et al., 2015). Our expectation, based on previous data obtained in other neurodegenerative disorders (reviewed in Fernández-Ruiz et al., 2007, 2015), was that activating the CB_2_ receptor, whose levels were found to be elevated in glial elements in the cerebellum of SCA patients (Rodríguez-Cueto et al., 2014a), may serve to attenuate glial reactivity and its associated inflammatory events. However, we did not find any evidence of CB_2_ receptor upregulation in the three CNS structures investigated in SCA-3 transgenic mice in our studies (Rodríguez-Cueto et al., 2016, 2017). As mentioned above, it is possible that the lack of response of the CB_2_ receptor derives from the poor reactive gliosis detected in these areas in SCA-3, a fact that had been found both in patients (Rüb et al., 2013) and has been previously described in the experimental model used in our studies (Silva-Fernandes et al., 2014) and confirmed in our studies (Rodríguez-Cueto et al., 2016, 2017).

## Author Contributions

CR-C and EL-P carried out the different experiments and analyses conducted in our laboratory and published previously, which were reviewed in this article. MG-R, MH-G, and JF-R designed and supervised these studies, whereas JF-R wrote the different drafts and the final version of this manuscript. All authors read, corrected, and approved this final version.

## Conflict of Interest Statement

The authors declare that the research was conducted in the absence of any commercial or financial relationships that could be construed as a potential conflict of interest.
